# Transcriptional Activation of Arabidopsis Zygotes Is Required for Initial Cell Divisions

**DOI:** 10.1038/s41598-019-53704-2

**Published:** 2019-11-20

**Authors:** Ping Kao, Michael D. Nodine

**Affiliations:** Gregor Mendel Institute (GMI), Austrian Academy of Sciences, Vienna Biocenter (VBC), Dr. Bohr-Gasse 3, 1030 Vienna, Austria

**Keywords:** Microscopy, Embryogenesis, Plant reproduction

## Abstract

Commonly referred to as the maternal-to-zygotic transition, the shift of developmental control from maternal-to-zygotic genomes is a key event during animal and plant embryogenesis. Together with the degradation of parental gene products, the increased transcriptional activities of the zygotic genome remodels the early embryonic transcriptome during this transition. Although evidence from multiple flowering plants suggests that zygotes become transcriptionally active soon after fertilization, the timing and developmental requirements of zygotic genome activation in *Arabidopsis thaliana* (Arabidopsis) remained a matter of debate until recently. In this report, we optimized an expansion microscopy technique for robust immunostaining of Arabidopsis ovules and seeds. This enabled the detection of marks indicative of active transcription in zygotes before the first cell division. Moreover, we employed a live-imaging culture system together with transcriptional inhibitors to demonstrate that such active transcription is physiologically required in zygotes and early embryos. Our results indicate that zygotic genome activation occurs soon after fertilization and is required for the initial zygotic divisions in Arabidopsis.

## Introduction

The transition of developmental control from parental-to-zygotic genomes is a pivotal event during animal and plant development. In animals, maternally inherited gene products regulate early embryogenesis while the zygotic genome remains transcriptionally quiescent until the maternal-to-zygotic transition (MZT). Two interdependent events constitute the MZT in animals: the degradation of inherited maternal gene products and zygotic genome activation (ZGA) when the zygotic genome breaks transcriptional quiescence to produce transcripts that instruct subsequent embryogenesis. While the MZT is universal in multiple species, the underlying mechanisms, scale and timing of ZGA are diverse (reviewed in ref.^1–4^). Investigating how different species evolved various mechanisms to initiate ZGA is crucial to understanding embryogenesis.

Compared to animals, the knowledge of timing and requirements of ZGA in flowering plants is limited. Histological and molecular evidence in *Hyacinthus orientalis* (hyacinth)^5,6^, *Nicotiana tabacum* (tobacco)^7,8^, *Oryza sativa* (rice)^9–13^, *Triticum aestivum* (wheat)^14,15^ and *Zea mays* (maize)^16–20^ altogether indicate that large-scale transcriptional activities increase in zygotes after fertilization and prior to the first division. These results suggest that, similar to animals, plant zygotic genomes may also transition from a transcriptionally quiescent to active state. However, plant and animal life cycles are fundamentally different, where plants alternate between haploid gametophytic and diploid sporophytic phases^21^. More specifically, a subset of sporophytic cells undergo meiosis to produce haploid spores, which divide mitotically to generate multicellular gametophytes containing eggs and sperms. Fertilization of the egg cell contained within each female gametophyte marks the onset of the sporophytic generation. Although it is unclear how similar the gametophytic-to-sporophytic transition in plants is to the MZT in animals, we have referred to the large-scale increase of transcriptional activities after fertilization as ZGA in the following text.

Although ZGA has been partially characterized in the model flowering plant *Arabidopsis thaliana* (Arabidopsis), the timing, parental contributions and requirements of ZGA was debatable. One model proposed that Arabidopsis zygotes are transcriptionally quiescent^22^ and early embryos mostly rely on maternal gene products for growth and division^23–27^. However, several mutants exhibiting defects in the initial asymmetric division of the zygote segregate in a recessive manner consistent with transcriptional activities of either parental allele being sufficient for the first zygotic division^28–35^. Moreover, transcriptome analyses indicated equal parental genomic contributions to the embryonic transcriptome as early as the 1-cell/2-cell stage^36^. Based on these results, it was proposed that the zygotic genome is activated within the first few hours after fertilization with equal contributions of maternal and paternal alleles to the transcriptome^36^. Although the maternal transcriptome dominance reported in a conflicting publication^25^ can be readily explained by the amount of maternal RNA contamination in the samples^37^, the precise timing and requirements of zygotic genome activation was unresolved until recently^38^. Here, we provide independent evidence by expansion microscopy and live-cell imaging to demonstrate that transcriptional activities are markedly increased soon after fertilization in Arabidopsis and that zygotic transcription is essential for the initial embryonic cell divisions.

## Results

### Expansion microscopy improves whole-mount fluorescent immunostaining

**Figure 1:**
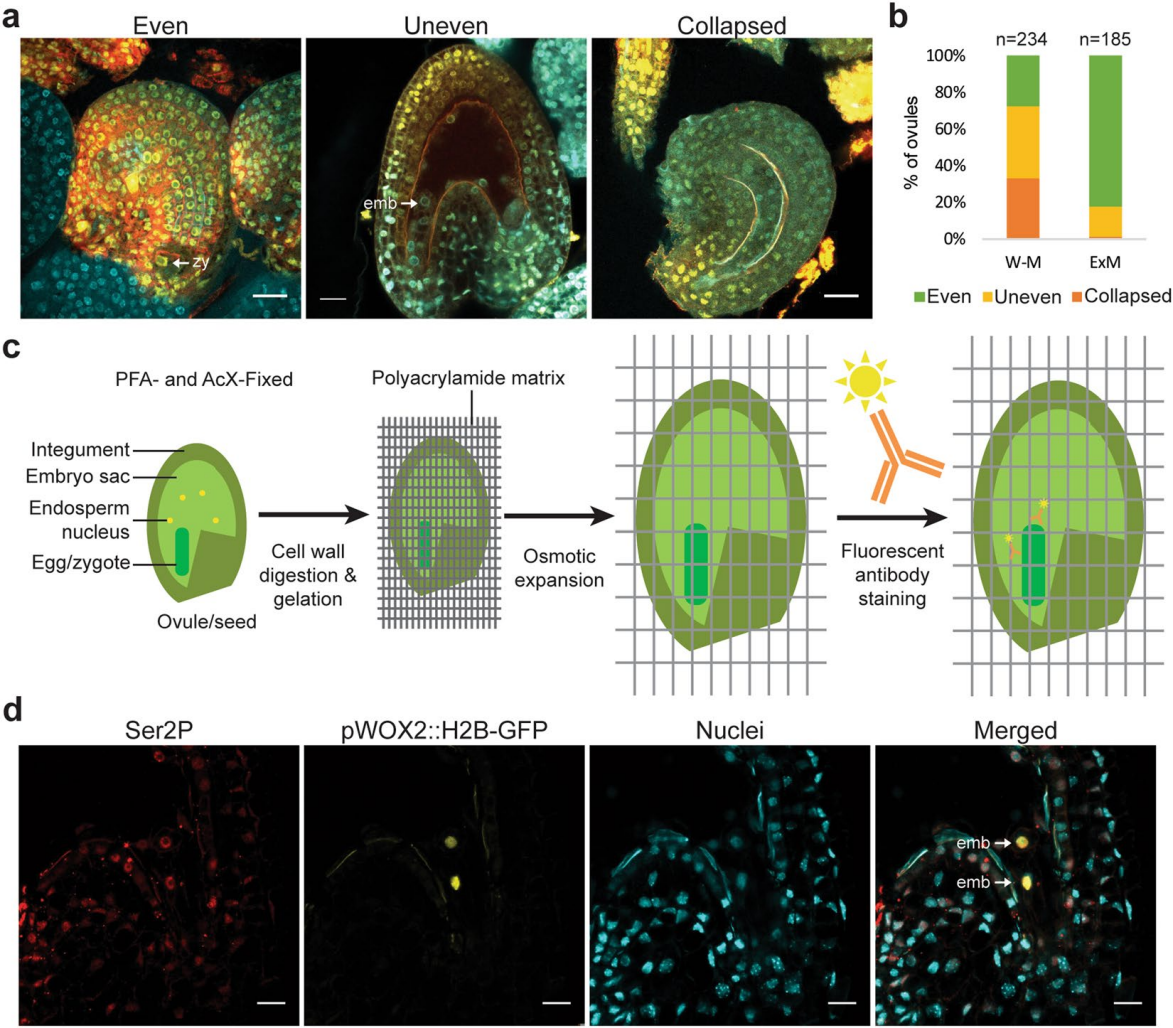
Expansion microscopy on Arabidopsis seeds. (**a)** Representative images of evenly stained samples (Even), unevenly stained samples (Uneven), or collapsed samples (Collapsed). Tubulin (red) and RNAPII Ser2P (yellow) were detected with immunofluorescence, and nuclei were stained with DAPI (cyan). **(b)** Quantification of the number of seeds with either even or uneven staining, or that were collapsed when using conventional whole-mount (W-H) or expansion microscopy (ExM) protocols. The total number of seeds examined with each method is indicated. **(c)** Schematic of expansion microscopy workflow. Ovules or seeds were fixed, incubated with cell wall digestion enzymes, embedded in a polyacrylamide gel matrix and osmotically expanded before fluorescent immunostaining (See Materials and Methods for details). **(d)** Protein retention after expansion in embryos. Immunofluorescent signal from DAPI-stained nuclei (cyan), RNAPII Ser2P (red) or HISTONE 2B-tagged GFP expressed with the embryo-specific WOX2 promoter (pWOX2::H2B-GFP; yellow) are shown. Scale bars represent 20 µm. zy, zygote; emb, embryo.

Phosphorylation of serine 2 on the carboxy-terminal domain of RNA polymerase II (RNAPII Ser2P) indicates elongating polymerase^39,40^. Therefore, we used conventional whole-mount fluorescent immunostaining^22,41^ on fertilized ovules (seeds) to detect evidence for RNAPII transcriptional activities in zygotes and embryos. We also stained against tubulin with antibodies and chromatin with 4′,6-diamidino-2-phenylindole (DAPI) to unambiguously identify egg and zygote nuclei because tubulin separates the zygote nucleus from surrounding endosperm nuclei. We obtained several samples with uniform and high signals, but found that the conventional protocol produced inconsistent results (Fig. 1a). Namely, 92/234 (39.3%) of samples exhibited uneven or no signal likely due to limited antibody accessibility (Fig. 1b). Embryos in particular had low signals because they were embedded within seeds. Moreover, 77/234 (32.9%) samples had collapsed embryo sacs, which contain the embryos (Fig. 1c), and thus were impossible to analyze (Fig. 1b). We therefore could not robustly detect RNAPII Ser2P with the conventional immunostaining protocol.

To improve the whole-mount fluorescent immunostaining method for Arabidopsis ovules and seeds, we adapted an expansion microscopy protocol (ExM)^42,43^. The ExM technique physically expands specimen uniformly in three dimensions to increase reagent accessibility and microscopic resolution while retaining the relative spatial positions of the signals. Samples were fixed in 4% paraformaldehyde and 0.1 mg/mL Acryloyl-X, SE (6-((acryloyl)amino)hexanoic acid, succinimidyl ester) (AcX), incubated with cell wall-digesting enzymes and embedded in an expandable polyacrylamide gel matrix (Fig. 1c). Samples were then expanded by osmotic pressure to improve antibody penetration and increase specimen size before immunostaining. We examined protein retention in expanded samples in seeds expressing a previously described embryo-specific reporter line (pWOX2::H2B-GFP, pWOX2::tdTomato-RCI2b)^44^. As expected, the nuclear-localized H2B-GFP signal was confined to embryonic nuclei indicating that our protocol successfully retained proteins in their proper subcellular localizations (Fig. 1d). We also stained against RNAPII Ser2P and found that the epitopes were retained and detectable with antibodies (Fig. 1d). To examine expansion ratios in three dimensions, we stained cell walls with SCRI Renaissance 2200 and nuclei with DAPI, and examined samples before and after expansion. Although expansions were not uniform in all three dimensions, ranging from 1.3 to 2.0 times in length depending on the experiment, the RNAPII Ser2P signal was high and consistent among samples. With the ExM technique we found that only 2/185 (1.1%) samples had collapsed embryo sacs and 31/185 (16.7%) had uneven staining, while 152/185 (82.2%) samples had high and consistent signal (Fig. 1b). Therefore, by physically expanding the specimens with the modified ExM technique before staining, we were able to make interior tissues more accessible to reagents. This method produced more consistent staining and enabled the robust detection of subcellular marks within zygotes.

### Visualization of transcriptional activities in eggs and zygotes

**Figure 2:**
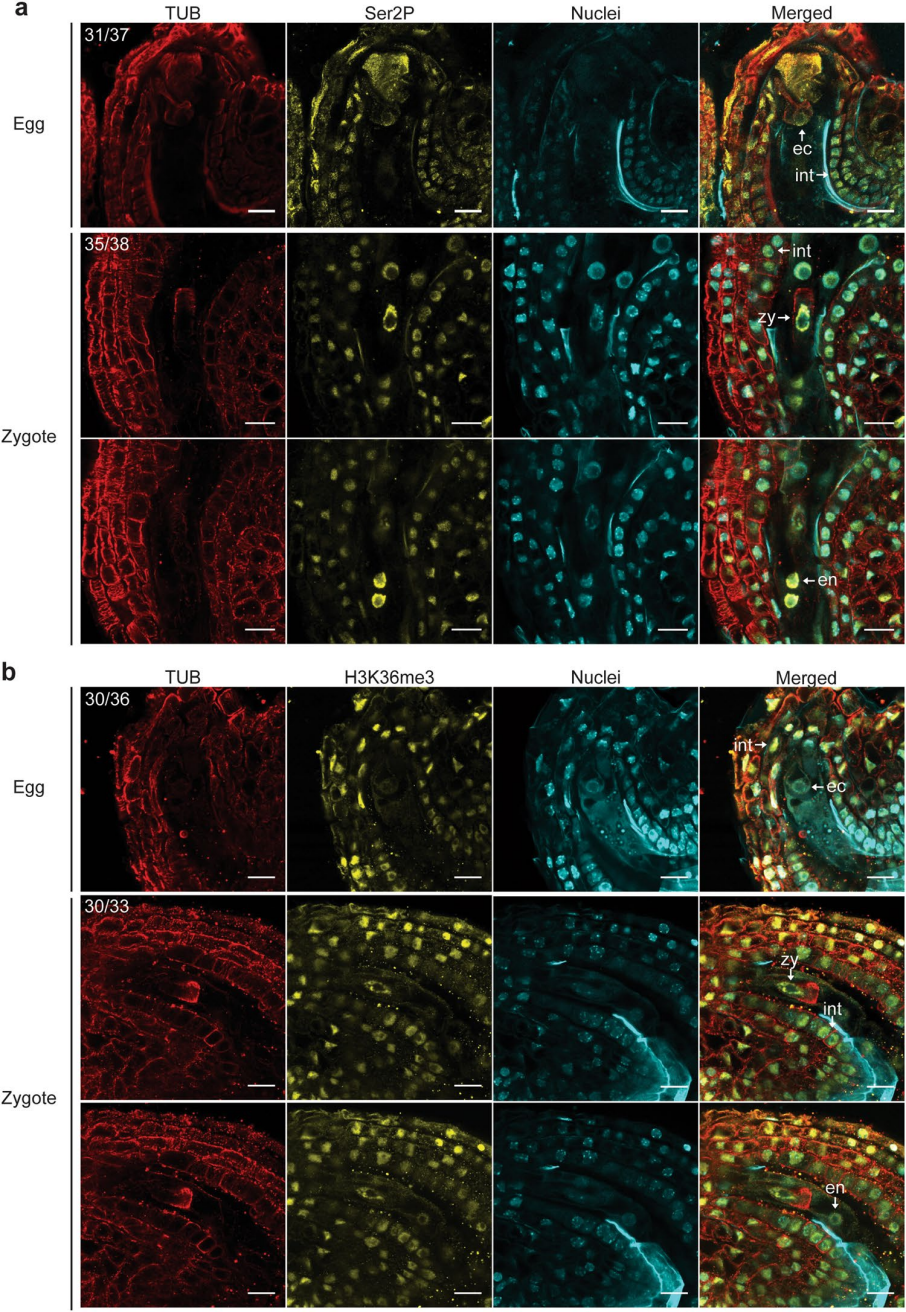
Visualization of transcriptional activities in eggs and zygotes. (**a**) Representative ExM images of tubulin (red), RNAPII Ser2P (yellow) and DAPI-stained nuclei (cyan) in ovules containing eggs (*top*) or seeds containing zygotes with focal planes on zygotes (*middle*) or endosperm (*bottom*). The number of ovules or seeds with similar staining patterns out of the total number examined are indicated. **(b)** Representative ExM images of tubulin (red), H3K36me3 (yellow) and nuclei (cyan) in ovules/seeds containing eggs (*top*) or zygotes with focal planes on zygotes (*middle*) or endosperm (*bottom*). The number of ovules or seeds with similar staining patterns out of the total number examined are indicated. Scale bars represent 20 µm. ec, egg cell; zy, zygote; en, endosperm; int, integument.

To infer transcriptional activities, we then employed the ExM approach described above to visualize RNAPII Ser2P in eggs and zygotes. We also stained against tubulin with antibodies and chromatin with DAPI to identify egg and zygote nuclei, and observed staining patterns that were consistent with previous reports^45,46^ (Supplementary Videos S1 and S2). In unfertilized ovules, the RNAPII Ser2P signal was low in 31 out of 37 (83.8%) of egg nuclei examined, but high in surrounding sporophytic integument nuclei (Fig. 2a). In contrast, after fertilization the RNAPII Ser2P signal was high in 35 out of 38 (92.1%) of zygote nuclei examined, as well as in surrounding endosperm and integument nuclei (Fig. 2a).

Trimethylation marks on histone H3 lysine 36 (H3K36me3) are deposited during RNAPII transcription elongation^47^. Therefore, we examined H3K36me3 levels in ovules and seeds using ExM to further inspect transcriptional activities in eggs and zygotes, respectively. Similar to RNAPII Ser2P, H3K36me3 signal was low in the majority of egg nuclei (30/36, 83.3%), but high in the majority of zygote nuclei (30/33, 90.9%), as well as in endosperm and surrounding sporophytic integument nuclei (Fig. 2b). The detection of H3K36me3 and RNAPII Ser2P in eggs and zygotes indicated that RNAPII transcription was relatively low in eggs and became highly active in zygotes after fertilization.

### Active transcription is physiologically required for zygote cell division

**Figure 3:**
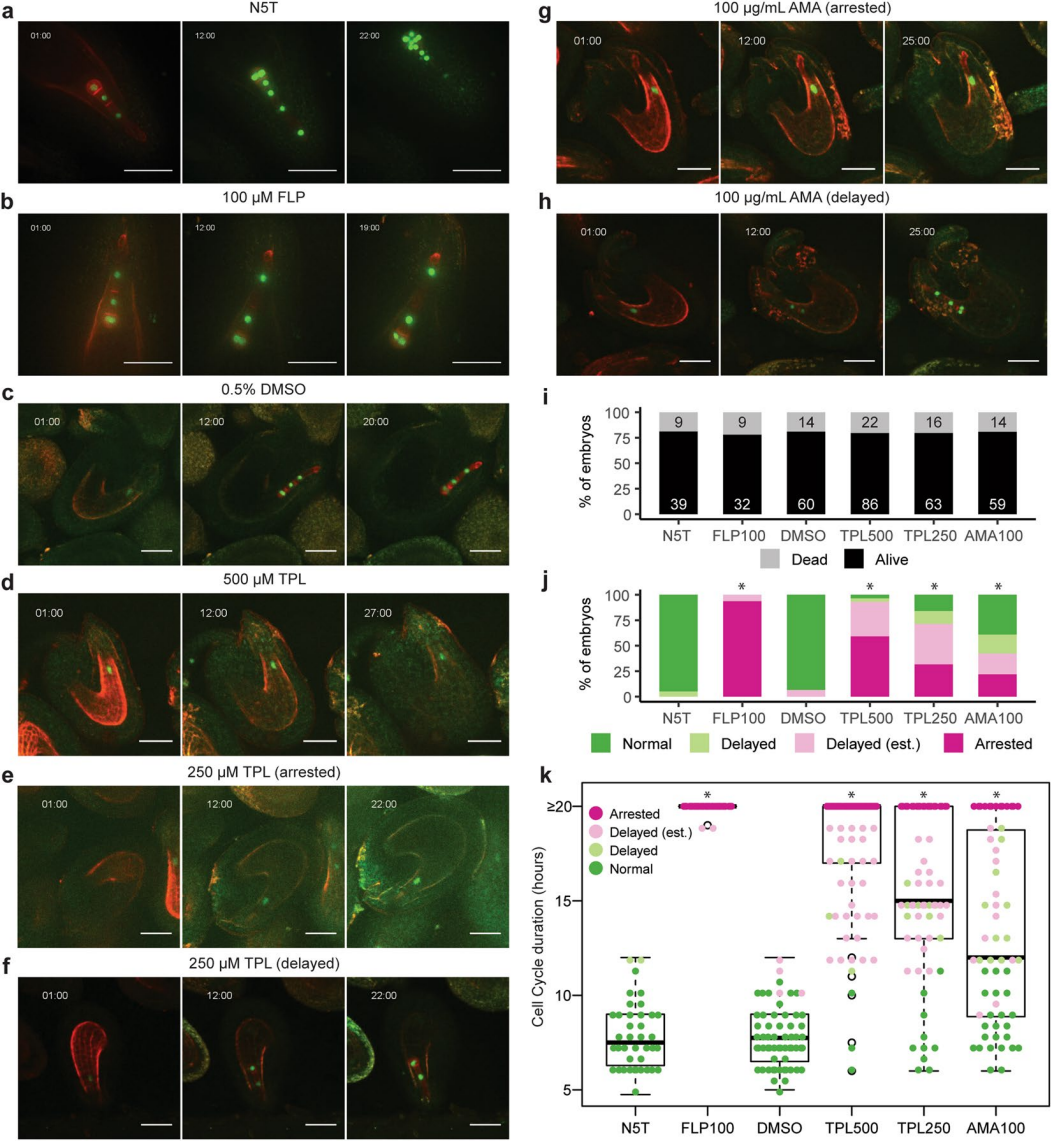
Transcription is required for initial embryonic cell divisions. **(a**–**h)** Time-lapse observations of embryos expressing pWOX2::H2B-GFP, pWOX2::tdTomato-RCI2b cultured in **(a)** N5T, **(b)** N5T with 100 µM FLP, **(c)** N5T with 0.5% DMSO, **(d)** N5T with 0.5% DMSO and 500 µM TPL, **(e**,**f)** N5T with 0.5% DMSO and 250 µM TPL or **(g**,**h)** N5T with 0.5% DMSO and 100 µg/mL AMA. The time after incubation is indicated as hours:minutes. **(i)** Bar chart illustrating the percentage of embryos that survived (Alive) or died (Dead) under various conditions. The number of dead and alive embryos are indicated in black and white, respectively. **(j)** Bar chart showing the percentage of embryos that divided normally or abnormally when cultured under various conditions. The total number of surviving embryos for each condition are indicated in Supplementary Table S1. Asterisks indicate *P*-values < 0.001 based on chi-squared tests that the observed differences in frequencies between test and control groups were related to different culture conditions. **(k)** Graph illustrating cell cycle durations for each culture condition based on the observed duration between cell divisions or the lower estimations on the duration (est.). Because of limitations on recording time, cell cycle durations for arrested embryos were set to an upper limit of 20 hours. The number of embryos examined for each condition are equal to the number of alive embryos labelled in (j), and the detail number of embryos for each phenotype are indicated in Supplementary Table S1. Each dot represents a biological replicate, thick horizontal bars indicate medians, and the top and bottom edges of the box indicate the 75th and 25th percentile, respectively. Asterisks indicate significantly different populations determined by two-sample Kolmogorov-Smirnov tests, *P*-values < 0.001. The mean and median cell cycle durations are indicated in Supplementary Table S1. Scale bars represent 50 µm. N5T, Nitsch medium with 5% Trehalose; FLP, flavopiridol; DMSO, dimethyl sulfoxide; TPL, triptolide; AMA, α-amanitin.

Classic transcriptional inhibition experiments in mouse^48^, *Drosophila melanogaster*^49^, *Caenorhabditis elegans*^50^, *Xenopus laevis*^51^ and *Danio rerio*^52^ early embryos elegantly demonstrated that inherited gene products were sufficient for early embryogenesis. Therefore, to test whether active transcription is required for Arabidopsis zygote development, we cultured seeds in the presence of transcriptional inhibitors and examined zygotic cell division patterns with live-imaging microscopy^44^. If inherited gene products were sufficient for early embryogenesis, then we expected no developmental delay or arrest when transcriptional inhibitors were included in the culture media. Seeds were cultured in the presence or absence of transcriptional inhibitors for one hour before acquiring the first image, and zygotes were labelled with nuclear-localized GFP and plasma membrane-localized tdTomato both of which were under the control of the embryo-specific WOX2 promoter (pWOX2::H2B-GFP, pWOX2::tdTomato-RCI2b)^44^. The nuclear-localized GFP signal can be observed in elongated zygotes before they divide and served as a marker specific to zygotes and early embryos. Similar to what was originally reported for this culture system^44^, we found that approximately 80% of embryos survived in our system under all conditions (Fig. 3i). That is, regardless of culture conditions approximately 20% of embryos lost fluorescent signal likely due to technical limitations of the system. We classified these embryos as “dead” and the remaining ones as “alive”, and only these surviving embryos were informative to test our hypothesis. We further categorized the alive embryos as “normal”, “delayed”, or “arrested” if the observed cell cycle duration was <12, ≥12 or ≥20 hours, respectively. Embryos with only one cell division observed were conservatively estimated to be delayed and categorized as “delayed (est.)”. As a positive control, we first compared cell division patterns of embryos cultured with or without the flavopiridol (FLP) kinase inhibitor, which causes cell-cycle arrest^53^. As expected, when cultured in the control Nitsch medium with 5% Trehalose (N5T) most (37/39, 94.9%) embryos divided normally similar to previous reports^44,46,54^ (Fig. 3a,j and Supplementary Video S3) with a 7.5-hour median cell cycle duration (Fig. 3k). In contrast, most embryos cultured with 100 µM FLP had arrested cell division (30/32, 93.8%; Fig. 3b,j and Supplementary Videos S4, *P*-value = 1.1 × 10^−14^, chi-squared test). Furthermore, the distribution of the cell cycle duration was significantly greater compared to N5T (Fig. 3k; *P*-value = 3.8 × 10^−15^, two-sample Kolmogorov-Smirnov test). This control test indicated that our system was capable of capturing cell cycle arrest in early embryogenesis.

Similar to N5T control media, most embryos within seeds cultured with 0.5% dimethyl sulfoxide (DMSO) divided normally (56/60, 93.3%; Fig. 3c,j and Supplementary Video S5; *P*-value = 0.99, chi-squared test) and had a 7.8-hour median cell cycle duration (*P*-value = 0.99, two-sample Kolmogorov-Smirnov test). Triptolide (TPL) induces protease-dependent degradation of RNAPII components^55,56^. We cultured seeds with TPL to test whether active transcription was required for zygotic divisions and early embryogenesis. When cultured with 500 µM TPL and 0.5% DMSO, 51/86 (59.3%) embryos were arrested, 32/86 (37.2%) had delayed cell divisions and only 3/86 (3.5%) developed normally (Fig. 3d,j and Supplementary Video S6). The proportion of embryos that were arrested or delayed was significantly greater than observed for the DMSO control condition (*P*-value = 8.8 × 10^−27^, chi-squared test), and the distribution of cell cycle duration was significantly greater compared to DMSO (Fig. 3k, *P*-value = 2.2 × 10^−16^, two-sample Kolmogorov-Smirnov test). Moreover, TPL had a dose-dependent effect on embryo development whereupon when seeds were cultured with 250 µM TPL, 20/63 (31.7%) embryos were arrested (Fig. 3e,j and Supplementary Videos S7), 33/63 (52.4%) had delayed divisions (Fig. 3f,j and Supplementary Videos S8) and 10/63 (15.9%) developed normally (*P*-value = 3.4 × 10^−17^ compared to DMSO control, chi-squared test). The distribution of cell cycle duration was significantly greater compared to DMSO but significantly less compared to 500 µM TPL (Fig. 3k, *P*-value = 2.2 × 10^−16^ and 1.5 × 10^−5^, respectively, two-sample Kolmogorov-Smirnov test).

To further test whether de novo transcription was required for zygote divisions and early embryogenesis, we also cultured seeds with α-amanitin (AMA), which binds to RNAPII and prevents nucleotide incorporation and transcript translocation^57,58^. In the presence of 100 µg/mL (108.8 µM) AMA, 23/59 (39.0%) embryos divided normally, while 13/59 (22.0%) were arrested (Fig. 3g,j and Supplementary Video S9) and 23/59 (39.0%) were delayed (Fig. 3h,j and Supplementary Video S10) in their development (*P*-value = 1.2 × 10^−9^ compared to DMSO control, chi-squared test). The distribution of cell cycle duration was significantly greater compared to DMSO (Fig. 3k, *P*-value = 1.6 × 10^−9^, two-sample Kolmogorov-Smirnov test) but similar to 250 µM TPL (*P*-value = 4.0 × 10^−3^, two-sample Kolmogorov-Smirnov test). Full details of the total number of samples examined and the cell cycle durations are listed in Supplementary Table S1. Although samples were cultured and imaged during different time spans due to technical reasons, the varying end time points do not affect our conclusions. As demonstrated by our results, the 20-hour time span is sufficient to capture at least two rounds of cell division for embryos cultured under control conditions. Because zygotes cultured with transcriptional inhibitors exhibited no or delayed zygotic division when monitored over longer periods of time compared to zygotes cultured under control conditions, this indicated that the delayed or arrested division was not due to insufficient observation time. The arrested and delayed cell cycles in the presence of the TPL and AMA transcriptional inhibitors indicated that de novo transcription was essential for the onset of zygote division and early embryogenesis.

## Discussion

Optimization of the ExM fluorescent immunostaining technique for seeds enabled the robust detection of RNAPII Ser2P and H3K36me3 in zygotes. Because RNAPII Ser2P and H3K36me3 are hallmarks of active transcription, this indicated that zygotes are transcriptionally active soon after fertilization and before the first division. Moreover, zygotes had arrested and delayed cell divisions when cultured in the presence of transcriptional inhibitors (i.e. TPL and AMA), which is consistent with ZGA being required for the first division. Our observations complement a recent transcriptome study demonstrating that ZGA occurs in zygotes and is required for elongation and division^38^.

While the conventional whole-mount immunostaining protocol can result in even-stained samples, we found that the method produced variable and inconsistent results. To improve the robustness of histological detection in Arabidopsis ovules and seeds, we adapted ExM for plant tissues in this study. Constraints imposed by undigested cytoskeleton and cell wall components limited the ability to fully expand samples. We omitted the proteinase K treatment described in the original ExM protocol because we found that even a mild proteinase K treatment (i.e. 5 U/mL for 30 minutes) resulted in signal loss without improving expansion ratios or consistency. Nevertheless, we were able to produce expanded samples with the ExM method, which makes interior tissues accessible to antibodies and resulted in more consistent immunostaining. Sample expansion also helped reduce autofluorescence, which is common in plant tissues. Our ExM protocol therefore provides a more robust and efficient option for conducting whole-mount fluorescent immunostaining of eggs and zygotes contained in ovules and seeds, respectively.

The detection of low levels of RNAPII Ser2P and H3K36me3 in mature eggs indicated that there is low transcriptional activity before fertilization. After fertilization, signal corresponding to RNAPII Ser2P and H3K36me3 was high demonstrating that transcriptional activities clearly increased in the zygote before the first division. It was previously reported that endosperm, but not zygote nuclei were transcriptionally active^22^. However, our observations indicate that both endosperm and zygote nuclei are transcriptionally active. The missing RNAPII Ser2P signal in zygotes in a previous report^22^ may be due to the antibodies used (H5, ab24758; Abcam, discontinued) or the inconsistency of the conventional whole-mount protocol. Our new histological evidence is consistent with a recent transcriptome study^38^ and indicates that ZGA occurs soon after fertilization in Arabidopsis.

As revealed by live-cell imaging, transcriptional inhibition resulted in delayed or arrested development and provided evidence that ZGA is required for initial zygotic division and early embryogenesis. The different responses of zygotes to TPL and AMA were likely due to the incubation timing because TPL and AMA are known to be fast-response and slow-response inhibitors, respectively^59^. The 250 µM TPL treatment, which is at least 100-fold greater than the recommended concentration for tissue culture^59^, showed more delayed than arrested zygote division. The requirement of high concentrations of transcriptional inhibitors to have a physiological effect may be due to the high transcriptional activities of zygotes, as well as the limited accessibility of transcriptional inhibitors to zygotes deeply-embedded within seeds. Consistent with our results using transcriptional inhibitors, previous RNAi-mediated knock-down of RNAPII resulted in delayed embryogenesis^22^. Although we cannot completely exclude that the transcriptional inhibition of ovule tissues supporting the developing embryo (e.g. integument and endosperm) are the primary cause of the embryo arrest we observed, it is clear that inherited parental transcripts are not sufficient and de novo transcripts are required for early embryogenesis regardless of their origin. Moreover, *in vitro* fertilized or isolated zygotes developed normally through early embryogenesis in the absence of surrounding maternal tissue in rice^60,61^, maize^62^ and tobacco^8,63^, and Arabidopsis embryos can develop to at least the globular stage in the absence of the endosperm^44,64^. These reports are consistent with the delayed and arrested zygotic division observed upon culturing with transcriptional inhibitors being primarily caused by the inability to transcribe genes in zygotes.

Because it is difficult to differentiate de novo transcribed from maternally inherited transcripts, the activation of the zygotic genome can be inferred from the transcriptional activities of paternal alleles. Several studies reported that early Arabidopsis embryos rely on maternal factors with little or no paternal activity^23,24,65–69^ and suggest a quiescent state in zygotes^22^. In contrast, other studies reported paternal allele expression in early embryos^70–75^. Additionally, mutants defective in the asymmetric division of the zygote were reported to be recessive, suggesting that both parental alleles are transcriptionally active after fertilization^28–35,76,77^. Moreover, a recent genome-wide study reported significant upregulation of 4,436 genes in zygotes compared to eggs^38^. Altogether these results indicate that genes involved in early embryogenesis are transcriptionally active in zygotes, and thus do not support gradual ZGA or reliance on maternal gene products during early Arabidopsis embryogenesis as previously proposed^26,27^.

Genetic, microscopic and genomic studies in additional flowering plants, including maize^16–20,78^, wheat^14,15^, tobacco^7,8,79^ and rice^9–13^, indicate that ZGA occurs soon after fertilization in zygotes of multiple flowering plant species. In plants, zygotes mark the transition from the haploid gametophytic to diploid sporophytic phase of the life cycle. Because there is no clear evidence of prolonged transcriptional quiescence after fertilization and parentally-inherited gene products are not sufficient for early embryogenesis, the transcriptome remodeling observed in plant zygotes during the gametophytic-to-sporophytic transition is fundamentally different than the maternal-to-zygotic transition in early animal embryos as previously proposed^80^. In further contrast to animals^2,3^, the apparent similarities in the timing of transcriptome remodeling across plant species is intriguing and may indicate similar underlying mechanisms shared among flowering plants. For example, maternally and paternally inherited gene products converge to rapidly activate *WUS HOMEOBOX 8 (WOX8)* gene expression in Arabidopsis zygotes^75,76,80,81^, and additional mechanisms integrating biparentally inherited information may exist in Arabidopsis and other plants. With published egg and zygote transcriptomes in multiple plants, as well as the increasing number of genome-wide approaches applicable to low amounts of input material, the field is poised to identify the key regulatory genes involved in transcriptome remodeling and initiation of embryogenesis.

## Materials and Methods

### Plant materials and growth conditions

*Arabidopsis thaliana* accession Columbia (Col-0) and pWOX2::H2B-GFP, pWOX2::tdTomato-RCI2b transgenic Col-0 plants were grown at 20–22 °C temperature and 16 h light/8 h dark cycles under incandescent lights (130–150 µmol/m^2^/s) in a climate-controlled growth chamber.

### Sample collection and staging

For egg-containing ovule collection, we carefully emasculated flower buds from stage 11–12^82^ under a dissection microscope and then kept in growth chamber for 24 hours. The emasculated pistils were then examined under a dissection microscope and only the pistils with no pollen on stigmatic papillae were collected for experiments. For zygote-containing seed collection, we selected self-pollinated siliques from stage 14–15^82^ and dissected the siliques as described in the following sections. The detailed staging were determined by morphology after image acquisition according to previous reports^44,46,75,83^.

### Cell wall digestion enzymes

We tested several cell wall digestion enzymes from multiple manufacturers and found the performance of the enzymes varied between manufacturers, as well as between batches from the same manufacturers. We tested driselase (Sigma), cellulase (Sigma), cellulase R10 (Duchefa, Yakult), cellulase RS (Duchefa, Yakult), pectolyase (Duchefa; discontinued), pectinase (Sigma), macerozyme R10 (Duchefa) and hemicellulase (Sigma) for conventional whole-mount protocol. We chose cellulase RS (Duchefa), hemicellulase (Sigma) and pectinase (Sigma) or macerozyme R10 (Duchefa) for expansion microscopy based on their performance and availability.

### Antibodies

We used primary antibodies detecting RNAPII Ser2P (ab5095, Abcam), H3K36me3 (ab9050, Abcam), tubulin (ab89984, Abcam), and secondary antibodies anti-rabbit-Alexa488 (ab150077, Abcam) and anti-chicken-Alexa555 (ab150170, Abcam) because these antibodies are commercially available, commonly used to detect corresponding epitopes^84–89^ and produced minimal non-specific signals as shown in Supplementary Video S11.

### Conventional whole-mount fluorescent immunostaining

Conventional whole-mount fluorescent immunostaining was performed according to a published protocol^41^. Seeds of self-fertilized Col-0 siliques at stages 14–15^82^ were isolated under a dissection scope. Isolated seeds were collected in 4% PFA, 0.1% Triton X-100 and 1× PBS solution. Seeds were then briefly vacuum infiltrated and incubated at room temperature for one hour. Fixed seeds were washed three times with 0.1% Triton X-100 and 1 × PBS before incubation with enzyme mix (1% driselase, 0.5% cellulase, and 1% pectolyase in water). Alternatively, seeds were washed once more with protoplast salt solution (20 mM MES, pH 5.0, 0.4 M mannitol, 20 mM KCl and 10 mM CaCl_2_) before incubation with protoplast enzyme solution (3% cellulase, 1% hemicellulase, 1% macerozyme, 20 mM MES, pH5.0, 0.4 M mannitol, 20 mM KCl and 10 mM CaCl_2_, 0.1% BSA and 1% β-mercaptoethanol) before use. Enzyme solution was prepared as previously described^90^. Seeds were incubated in either enzyme solution at 37 °C for 2 hours with gentle agitation. Digested seeds were washed twice with 0.2% Triton X-100, 1× PBS and embedded in 3% polyacrylamide matrix on adhesive slides as described^41^. Slides were incubated with 1% Triton X-100, 1× PBS at 4 °C for two hours with gentle agitation. Permeabilized samples were incubated with 1× PBS, 2% BSA, 0.1% Triton X-100 at room temperature for one hour. Samples were then incubated with 1× PBS, 2% BSA, 0.1% Triton X-100 and 1:500 dilution of primary antibodies against RNAPII Ser2P (ab5095, Abcam) and tubulin (ab89984, Abcam) at 4 °C overnight with gentle agitation. On the next day, samples were washed with 0.2% Triton X-100, 1× PBS at 4 °C for one hour at least five times. For secondary antibody incubation, samples were incubated in 1:500 dilution of anti-rabbit-Alexa488 (ab150077, Abcam) and anti-chicken-Alexa555 (ab150170, Abcam) in 1× PBS, 2% BSA, 0.1% Triton X-100 at 4 °C overnight with gentle agitation. Samples were then washed with 10 µg/mL 4′,6-diamidino-2-phenylindole (DAPI), 0.2% Triton X-100, 1× PBS at 4 °C in the dark for one hour twice and washed three times without DAPI. Samples were mounted in Vectashield Mounting Medium (H-1200, Vector) and imaged by ZEISS LSM700/780 with 25× oil objective at maximal resolution (>1024 × 1024) as 8-bit images at 13 z-stacks with 1 µm intervals by ZEN software. DAPI signals were excited by 405 nm laser and passed through SP490 filters. Alexa488 signals were excited by 488 nm laser and passed through BP490-635 filters. Alexa555 signals were excited by 555 nm laser and passed through 560–1000 nm filters. Pinhole sizes were kept as 1 airy unit for each color, and color channels were scanned separately to avoid false signal.

### Expansion microscopy

We modified published ExM protocols for plant tissues^42,43^. For each condition, the immunostainings were conducted at least three times and are considered technical replicates. For each technical replicate ≥10 ovules or seeds from 1-2 flowers on ≥4 individual plants were recorded as biological replicates. Pistils containing unfertilized ovules or siliques containing fertilized seeds were carefully sliced open longitudinally under a dissection scope and transferred to 1× PBS, 0.1% Triton X-100, 4% PFA and 0.1 mg/mL Acryloyl-X, SE (6-((acryloyl)amino)hexanoic acid, succinimidyl ester; Thermo Fisher). After brief vacuum infiltration, samples were incubated at 4 °C overnight as recommended by a previous report^43^. Samples were washed with water twice and then washed once more with protoplast salt solution (20 mM MES, pH5.0, 0.4 M mannitol, 20 mM KCl and 10 mM CaCl_2_) before incubation with protoplast enzyme solution (3% cellulase, 1% hemicellulase, 1% macerozyme, 20 mM MES, pH 5.0, 0.4 M mannitol, 20 mM KCl and 10 mM CaCl_2_, 0.1% BSA and 1% β-mercaptoethanol) before use^90^. Samples were incubated at 37 °C for 2–3 hours (depending on developmental stage) with gentle agitation. Cell wall-digested samples were washed twice with 0.2% Triton X-100, 1× PBS and then permeabilized with 1% Triton X-100, 1× PBS at 4 °C for two hours. Pistils or siliques were then carefully dissected on depression slides under a dissection scope. Ovules/seeds attached to septums (i.e. ovule/seed strings) were detached from pistils/siliques and transferred to 0.2% Triton X-100, 1× PBS. Isolated ovule/seed strings were drained briefly and incubated in monomer solution (1× PBS, 2 M NaCl, 8.625% (w/w) sodium acrylate, 2.5% (w/w) acrylamide, 0.15% (w/w) N,N′-methylenebisacrylamide) at 4 °C overnight. Samples were then polymerized with 0.2% ammonium persulfate (APS) and tetramethylethylenediamine (TEMED) on glass slides with an adequate spacer (200–300 µm) at room temperature for one hour. Proteinase K treatment was omitted because it resulted in massive loss of epitopes. Gel slices containing ovule/seed strings were removed with razor blades and every two slices were transferred to 1 mL of water in 2 mL microtubes for expansion for one hour. The expansion was repeated at least twice. Expanded samples were then immunostained as described above for conventional immunostaining. For image acquisition, stained samples were mounted in 0.2% Triton X-100, 1× PBS with Frame-Seal™ (BIO-RAD, SLF0601) and imaged by ZEISS LSM700/780 with 25× oil objective at maximal resolution (>1024 × 1024) as 8-bit images at 13 z-stacks with 1 µm intervals by ZEN software. DAPI signals were excited by 405 nm laser and passed through SP490 filters. Alexa488 signals were excited by 488 nm laser and passed through BP490-635 filters. Alexa555 signals were excited by 555 nm laser and passed through 560–1000 nm filters. Pinhole sizes were kept as 1 airy unit for each color, and color channels were scanned separately to avoid false signal.

### Live-cell imaging

The ovule culture was performed as previously reported with slight modifications^44,46^. For each condition we performed ≥ 3 technical replicates where we collected ≥100 self-pollinated seeds at stage 14–15^82^ from four individual plants. Seeds were carefully isolated from siliques under a dissection scope and transferred to N5T medium (5% trehalose dihydrate (Sigma), 1× Nitsch basal salt mixture (Duchefa), 1× Gamborg’s vitamin solution (Sigma), 0.05% MES-KOH, pH 5.8). A brief vacuum infiltration was applied to seeds followed by a 30-minute incubation in N5T medium or medium supplemented with α-amanitin (Sigma), flavopiridol (Sigma), triptolide (Cayman Chemical), or dimethyl sulfoxide (Fisher Scientific) at room temperature with gentle agitation to submerge seeds completely. The seeds were then transferred to micro-Insert 4 Well in µ-Dish (ibidi) or µ-Slide 8 Well (ibidi) with corresponding medium for live-cell imaging. Time series images were recorded at 10 z-stacks with 1 µm intervals by Yokogawa CSU X1 spinning disc and Axio Observer (ZEISS) with 40× oil objective, Hamamatsu EMCCD 9100-13 camera and ZEN software, or Visiscope Spinning Disc Confocal (Visitron Systems GmbH) with 20× air objective, Andor Ixon Ultra 888 EMCCD camera and Visiview software. GFP was excited by 488 nm laser and emitting signals were passed through 525/550 nm filters. tdTomato was excited by 561 nm laser and resulting signals were passed through 605/670 nm filters. The first images were taken one hour after incubation and the time-lapsed images were taken every 30 to 60 minutes for at least 20 hours.

### Image processing

The eggs and zygotes were distinguished according to their morphology as previously described^44,46,75,83^. More specifically, eggs had an average length of 20–25 µm and nuclei polarized to the chalazal end, while the zygotes elongated to approximately 70 µm before dividing and the nuclei were apically localized approximately one-third from the chalazal end during elongation. We considered each egg or zygote as a biological replicate. All confocal microscope images were slightly adjusted for brightness by ZEN software to nearly saturate the signals in integument nuclei for qualitative comparison before exporting and scale bars were added by Fiji according to recorded pixel sizes. Spinning disc microscope images were processed by Fiji. For each timepoint the z-stacks were merged by maximum intensity projection, and then the contrast and brightness were adjusted for each channel. This procedure was necessary because the raw signals were kept to minimal levels to avoid phototoxicity. Color channels were then merged and images were cropped before adding scale bars and time stamps. For live-cell imaging experiments, the zygote/embryo phenotypes were determined according to the following criteria. “Normal” embryos had no morphological defects and <12 hours between cell divisions consistent with previous observations^44^. “Dead” zygotes/embryos exhibited cell shrinkage or loss of fluorescent signals indicative of cell death. Otherwise, we considered the zygotes/embryos as “arrested” or “delayed” if there was either no cell division for >20 hours or the duration of the cell-cycle was 12–20 hours, respectively. When only one cell division was observed within the recorded videos, zygotes/embryos were conservatively estimated to be delayed “delayed (est.)” instead of “arrested” and the recorded cell-cycle durations were underestimated. To minimize potential bias, all movies were examined and classified by a person who did not perform the experiment and had no knowledge about their identities.

### Statistics

The numbers of each phenotype for each condition (Supplementary Table S1) were used to perform chi-squared tests to determine if there was a significant difference between the frequencies observed in test groups (FLP100, TPL500, TPL250 or AMA100) compared to control groups (N5T or DMSO). The recorded cell cycle durations (Fig. 3k) were used to perform two-sample one-tailed Kolmogorov-Smirnov tests to determine if the distribution of cell cycle duration in a test condition was greater than or similar to a reference condition. R version 3.6.1 was used to perform all statistical tests with default settings and to generate charts presented in Fig. 3.

## Supplementary information

Supplementary Materials Document

Supplementary Video S1

Supplementary Video S2

Supplementary Video S3

Supplementary Video S4

Supplementary Video S5

Supplementary Video S6

Supplementary Video S7

Supplementary Video S8

Supplementary Video S9

Supplementary Video S10

Supplementary Video S11
