# Supplementary material for: Transcriptional Activation of Arabidopsis Zygotes Is Required for Initial Cell Divisions
Source: Sci Rep. 2019 Nov 20;9:17159. doi: 10.1038/s41598-019-53704-2 (PMC6868190; doi:10.1038/s41598-019-53704-2)
Supplement: Supplementary file 1 — Supplementary Materials Document [file 41598_2019_53704_MOESM1_ESM.docx]

**Supplementary Information**

Transcriptional Activation of Arabidopsis Zygotes Is Required for Initial Cell Divisions

Ping Kao^1^ and Michael Nodine^1,*^

^1^Gregor Mendel Institute (GMI), Austrian Academy of Sciences, Vienna Biocenter (VBC), Dr. Bohr-Gasse 3, 1030 Vienna, Austria

*Corresponding Author: [michael.nodine@gmi.oeaw.ac.at](mailto:michael.nodine@gmi.oeaw.ac.at)

**LEGENDS FOR SUPPORTING INFORMATION**

**Supplementary Figure S1.** Additional panel of images for Figure 2.

**Supplementary Table S1.** Sample number and cell cycle duration for live-cell imaging.

**Supplementary Video S1.** Unadjusted z-stack series of fluorescent immunostaining on expanded zygote samples (Example 1). The scale bar represents 20 µm. Staining on nuclei, RNAPII Ser2P and tubulin are presented in cyan, yellow and red, respectively.

**Supplementary Video S2.** Unadjusted z-stack series of fluorescent immunostaining on expanded zygote samples (Example 2). The scale bar represents 20 µm. Staining on nuclei, RNAPII Ser2P and tubulin are presented in cyan, yellow and red, respectively.

**Supplementary Video S3.** Live-cell imaging of embryos cultured in N5T medium. The scale bar represents 50 µm.

**Supplementary Video S4.** Live-cell imaging of embryos cultured with 100 µM FLP in N5T medium. The scale bar represents 50 µm.

**Supplementary Video S5.** Live-cell imaging of embryos cultured with 0.5% DMSO in N5T medium. The scale bar represents 50 µm.

**Supplementary Video S6.** Live-cell imaging of embryos cultured with 500 µM TPL, 0.5% DMSO in N5T medium. The scale bar represents 50 µm.

**Supplementary Video S7.** Live-cell imaging of embryos cultured with 250 µM AMA, 0.5% DMSO in N5T medium with arrested zygotes. The scale bar represents 50 µm.

**Supplementary Video S8.** Live-cell imaging of embryos cultured with 250 µM AMA, 0.5% DMSO in N5T medium with arrested zygotes. The scale bar represents 50 µm.

**Supplementary Video S9.** Live-cell imaging of embryos cultured with 100 µg/mL AMA, 0.5% DMSO in N5T medium with arrested zygotes. The scale bar represents 50 µm.

**Supplementary Video S10.** Live-cell imaging of embryos cultured with 100 µg/mL AMA, 0.5% DMSO in N5T medium with delayed division. The scale bar represents 50 µm.

**Supplementary Video S11.** Unadjusted z-stack series of fluorescent immunostaining without primary antibodies. The scale bar represents 20 µm. Staining on nuclei, anti-rabbit-Alexa488 and anti-chicken-Alexa555 are presented in cyan, yellow and red, respectively.


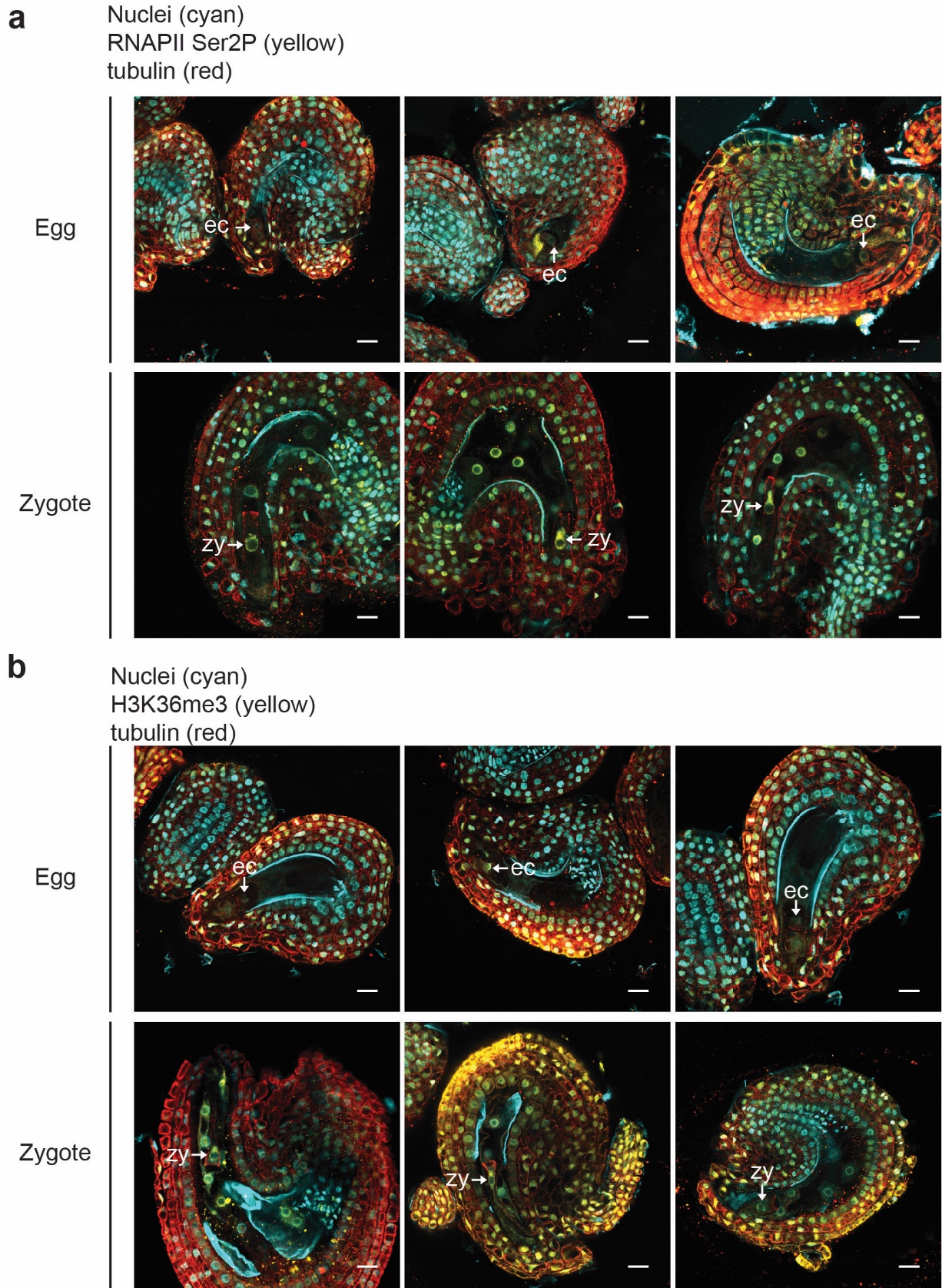


**Supplementary Figure S1. Visualization of transcription activities in eggs and zygotes.** **(a)** Unadjusted expansion microscopy images showing one focal plane of staining against tubulin (red), RNAPII Ser2P (yellow) and DAPI-stained nuclei (cyan) in ovules containing eggs (top) or seeds containing zygotes (bottom). **(b)** Unadjusted expansion microscopy images showing one focal plane of staining against tubulin (red), H3K36me3 (yellow) and DAPI-stained nuclei (cyan) in ovules containing eggs (top) or seed containing zygotes (bottom). Scale bars represent 20 µm. ec, egg cell; zy, zygote.

| **Supplementary Table S1.** Number of samples examined in live-cell imaging and the cell cycle durations (c.c). The number of embryos presented in Figs. 3i, 3j and 3k are listed, as well as the mean and median cell cycle durations observed. The delayed (est.) indicates the lower estimation of cell cycle duration because only one cell division was observed during recording. c.c, cell cycle duration; hr, hour. | | | | | | | |
| --- | --- | --- | --- | --- | --- | --- | --- |
| Conditions | | N5T | + 100 µM FLP | + 0.5% DMSO | + 500 µM TPL | + 250 µM TPL | + 100 µg/mL AMA |
| Labels | | N5T | FLP100 | DMSO | TPL500 | TPL250 | AMA100 |
| # of embryos | Dead | 9 | 9 | 14 | 22 | 16 | 14 |
|  | Arrested | 0 | 30 | 0 | 51 | 20 | 13 |
|  | Delayed (est.) | 0 | 2 | 4 | 29 | 25 | 12 |
|  | Delayed | 2 | 0 | 0 | 3 | 8 | 11 |
|  | Normal | 37 | 0 | 56 | 3 | 10 | 23 |
|  | Alive | 39 | 32 | 60 | 86 | 63 | 59 |
|  | Total | 48 | 41 | 74 | 108 | 79 | 73 |
| c.c (hr) | mean | 7.85 | 19.94 | 7.84 | 17.92 | 15.24 | 13.28 |
|  | median | 7.5 | 20 | 7.75 | 20 | 15 | 12 |
